# Anticancer Effect of Cold Atmospheric Plasma in Syngeneic Mouse Models of Melanoma and Colon Cancer

**DOI:** 10.3390/molecules28104171

**Published:** 2023-05-18

**Authors:** Joon-Min Jung, Hae-Kyeong Yoon, Su-Yeon Kim, Mi-Ra Yun, Gyeong-Hoon Kim, Woo-Jin Lee, Mi-Woo Lee, Sung-Eun Chang, Chong-Hyun Won

**Affiliations:** 1 Department of Dermatology, Asan Medical Center, University of Ulsan College of Medicine, 88 Olympic-ro 43-gil, Songpa-gu, Seoul 05505, Republic of Korea; 2 Asan Institute for Life Sciences, 88 Olympic-ro 43-gil, Songpa-gu, Seoul 05505, Republic of Korea

**Keywords:** cold atmospheric plasma, cancer treatment, melanoma, colon cancer, syngeneic mouse model

## Abstract

Cold atmospheric plasma (CAP) may have applications in treating various types of malignant tumors. This study assessed the anticancer effects of CAP using melanoma and colon cancer cell lines. CAP treatment significantly reduced the in vitro viability of melanoma and colon cancer cell lines and had a negligible effect on the viability of normal human melanocytes. Additionally, CAP and epidermal growth factor receptor (EGFR) inhibitor had an additive anticancer effect in a CAP-resistant melanoma cell line. Reactive oxygen and nitrogen species known to be generated by CAP enhanced the anticancer effects of CAP and EGFR inhibitors. The in vivo anticancer activities of CAP were evaluated by testing its effects against syngeneic tumors induced in mice by melanoma and colon cancer cells. CAP treatment reduced tumor volume and weight in both cancer models, with the extent of tumor reduction dependent on the duration and number of CAP treatments. Histologic examination also revealed the tumoricidal effects of CAP in both tumor models. In conclusion, CAP inhibits the growth of mouse melanoma and colon cancer cell lines in vitro and shows tumoricidal effects against mouse models of melanoma and colon cancer in vivo.

## 1. Introduction

The medical applications of cold atmospheric plasma (CAP) include skin decontamination, tooth bleaching, wound healing, and cancer therapy [1,2]. CAP has a significant impact on cellular biology, primarily by inducing the production of reactive oxygen species (ROS) [3], which act as second messengers in redox biology and can induce cellular differentiation and structural changes [4,5]. ROS produced by CAP can damage cellular DNA and lead to apoptosis [6]. Treatment of cancer cell lines with CAP in vitro effectively inhibits cell growth without resistance [7]. Furthermore, exposing cancer cells to CAP in vitro reduced cell migration and induced cellular apoptosis [8,9]. Cancer cells may be more vulnerable to CAP exposure than nonmalignant cells, indicating that CAP can selectively reduce the number of cancer cells with less destructive effects on adjacent nonmalignant cells [10,11,12]. This selective effect may depend on baseline intracellular ROS, aquaporin expression, or the cholesterol composition of the cell membrane [13,14]. CAP may also activate immune responses to tumor cells, inducing immune-mediated tumor cell death and enhancing macrophage function [15,16]. Furthermore, CAP reportedly has selective anti-tumor effects against oral squamous cell carcinoma by triggering nitric oxide (NO)-induced dysfunction of epidermal growth factor receptor (EGFR) [17]. This potential selectivity of CAP toward cancer cells suggests it may be a promising new treatment modality in oncology. For example, CAP can be applied to unresectable tumors in critical anatomical areas, with minimal potential risk of unintended local destruction of normal structures.

Several in vivo studies and clinical trials have evaluated the efficacy and safety of CAP in cancer treatment [7]. However, optimizing these effects requires comparisons of different types of CAP across diverse tumor types. Additional studies are required to objectively evaluate the role of CAP in cancer therapeutics and to design reliable treatment protocols.

Melanoma is a potentially fatal cutaneous malignancy. Although immunotherapy, including immune checkpoint inhibitors, has demonstrated the possibility of long-term survival in patients with advanced-stage melanoma, it is often resistant to various medical therapies [18,19,20,21]. The easy application of CAP to melanomas, most commonly located on the skin, highlight it as an appealing alternative when tumors, including adequate margins, cannot be completely resected. Additionally, the refractoriness to treatment of advanced melanoma suggests the need for new combination therapies. Thus, CAP, which can be readily combined with other systemic treatments such as EGFR inhibitors, is an attractive option. Several receptor tyrosine kinases, including members of the EGFR family, are found on the surface of melanoma cells. Although the role and clinical significance of their expression are not fully understood, EGFRs may play a role in the progression and metastasis of a subset of melanomas [22]. Therefore, targeting EGFR could also be a therapeutic option for patients with these tumors [23]. In addition, recent developments in plasma sources have made possible the propagation of CAP through a flexible endoscope [24], allowing CAP to be used to treat tumor types such as colon cancer.

This study was designed to evaluate the ability of direct CAP treatment to inhibit the growth of melanoma and colon cancer cells in vitro and to reduce targeted tumor volumes in vivo using syngeneic mouse models. In addition, the possible therapeutic role of combined treatment with CAP and EGFR inhibitors was assessed in melanomas.

## 2. Results

### 2.1. Selective Anticancer Effect of Cold Atmospheric Plasma

**Figure 1:**
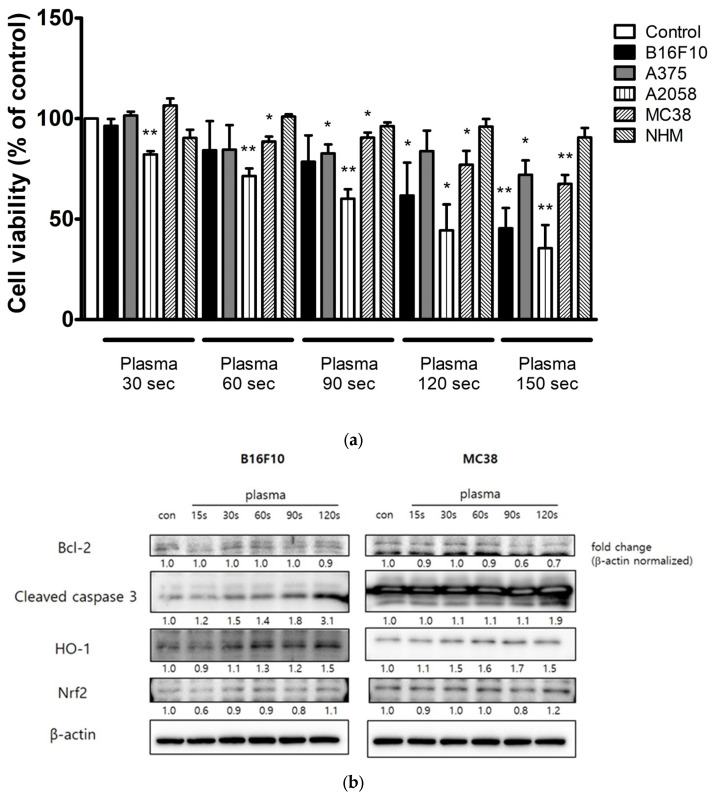
(**a**) Effects of cold atmospheric plasma (CAP) on the viability of B16F10 murine melanoma cells, A375 human melanoma cells, A2058 human melanoma cells, MC38 murine colon cancer cells, and normal human melanocytes. Cell viability was measured 24 h after treatment with CAP for the indicated times (30, 60, 90, 120, or 150 s). Exposure to CAP effectively reduced cancer cell viability in a treatment-duration-dependent manner. Data represent the mean ± standard deviation of three separate experiments. * *p* < 0.05, ** *p* < 0.01 compared with untreated control cells. (**b**) Western blot analysis of the expression levels of Bcl-2, cleaved caspase 3, heme oxygenase-1 (HO-1), Nrf2, and β-actin 24 h after treatment of B16F10 and MC38 cells with cold atmospheric plasma (CAP) for the indicated times. Levels of protein expression were quantified by densitometry after normalization to the optical density of β-actin. Treatment with CAP upregulated the expression level of HO-1 in B16F10 and MC38 cancer cells, while the expression level of cleaved caspase-3 was increased by CAP in B16F10 cancer cells.

The anticancer effects of CAP on the murine melanoma cell line B16F10, the human melanoma cell lines A375 and A2058, and the murine colon cancer cell line MC38 were investigated by applying CAP for up to 150 s and measuring cell viability after 24 h. Normal human melanocytes (NHMs) were treated with CAP for up to 150 s to assess whether CAP had selective effects on cancer cells. CAP treatment significantly reduced the viability of each cancer cell line in a treatment-duration-dependent manner but had a negligible effect on the viability of NHMs (Figure 1a). These results suggest that CAP has more potent activity against malignant than nonmalignant cell lines; however, the degree of response varied. CAP had a lower effect on the viability of A375 cells than on the other cancer cell lines.

The cleaved, activated form of caspase-3 is an indicator of the activation of pro-apoptotic signaling pathways [25], whereas heme oxygenase-1 (HO-1) expression is induced by oxidative stress and reported to mediate apoptosis [26]. Treatment of B16F10 and MC38 cancer cells with CAP upregulated the levels of cleaved caspase-3 or HO-1 expression (Figure 1b), suggesting that CAP treatment induces apoptosis in cancer cell lines through a mechanism involving oxidative stress. The expression levels of Bcl-2 and Nrf2 did not increase through CAP treatment in the cancer cell lines (Figure 1b).

### 2.2. Cold Atmospheric Plasma and Epidermal Growth Factor Tyrosine Kinase Inhibitor Have an Additive Antimelanoma Effect

**Figure 2:**
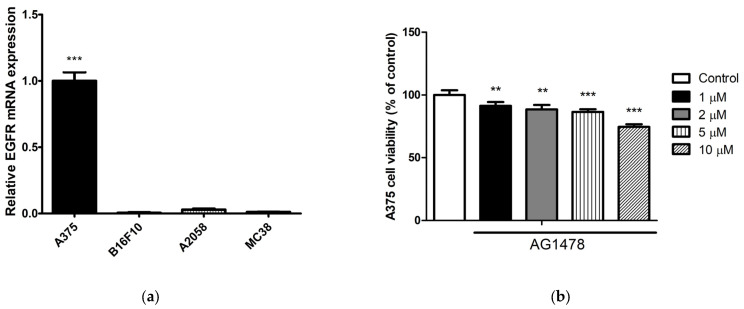

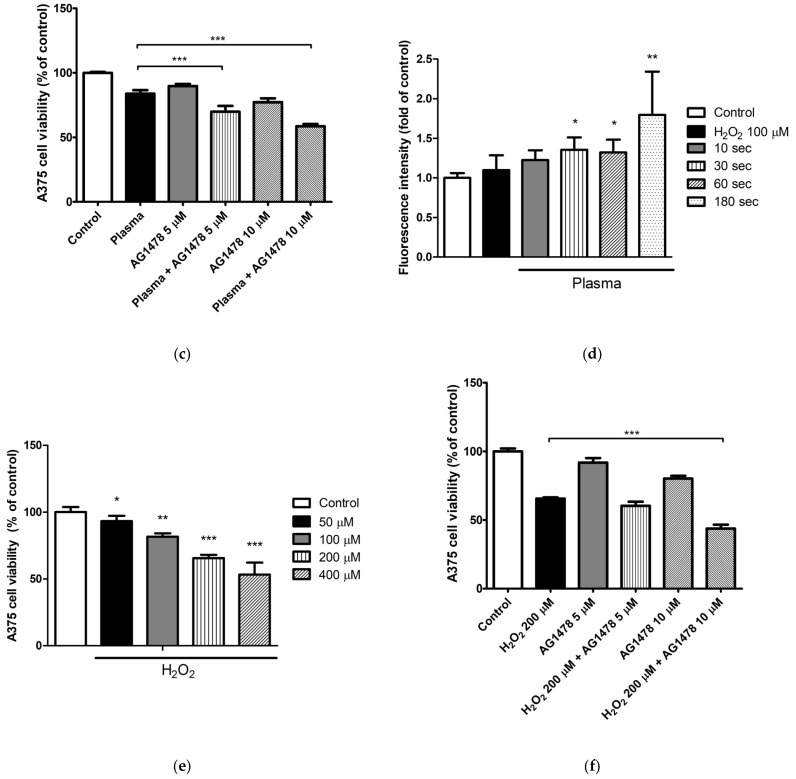

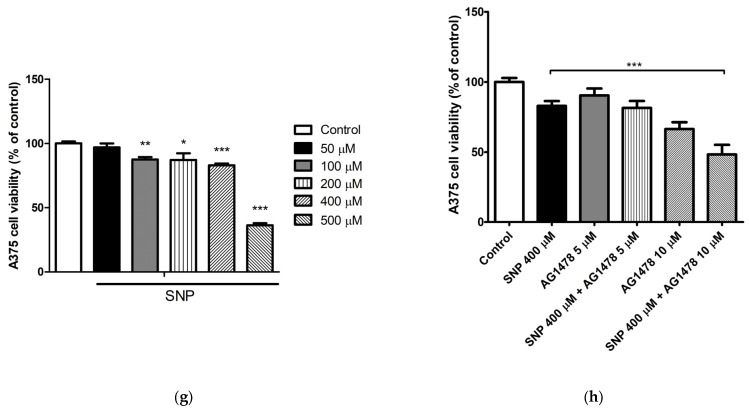
Additive antimelanoma effects of AG1478, a selective inhibitor of epidermal growth factor receptor (EGFR), and cold atmospheric plasma (CAP). (**a**) Relative expression levels of EGFR mRNA in A375, B16F10, A2058, and MC38 cells. (**b**) Viability of A375 cells 24 h after treatment with AG1478 at indicated concentrations. (**c**) A375 cells were pretreated with AG1478 at indicated concentrations 1 h before CAP treatment for 120 sec, and cell viability was assessed 24 h after the CAP treatment. (**d**) Fluorescence intensity for reactive oxygen species was detected using a microplate reader. The intensity increased as the treatment duration increased to 3 min. (**e**,**g**) Viability of A375 cells 24h after treatment with H_2_O_2_ or SNP at indicated concentrations. (**f**,**h**) A375 cells were pretreated with AG1478 at indicated concentrations (5 μM or 10 μM) 1 h before treatment with H_2_O_2_ (200 μM) or SNP (400 μM), with cell viability assessed 24 h after treatment. Data represent the mean ± standard deviation of three separate experiments. * *p* < 0.05, ** *p* < 0.01, *** *p* < 0.001.

Given the relative resistance of A375 cells to CAP, and the greater abundance of EGFR mRNA expressed in these cells compared with other cell lines (Figure 2a), we explored whether an EGFR inhibitor, alone or combined with CAP, had additive antimelanoma effects in A375 cells. When A375 cells were treated with various concentrations of AG1478, a selective inhibitor of EGFR, cell viability decreased in a similar manner for concentrations below 5 µM (Figure 2b). Therefore, for subsequent experiments, we used concentrations of 5 µM and 10 µM. When A375 cells were treated with a combination of an EGFR inhibitor and CAP, an additive anticancer effect was observed (Figure 2c). These results suggest that a combination of a systemic EGFR inhibitor and local CAP administration could be a potential therapeutic option for patients with melanoma.

CAP generates various effectors, including ROS, reactive nitrogen species (RNS), free-charged particles, radicals, ultraviolet radiation, and electric fields [1,3,12]. The main reactive species produced by the CAP system we adopted are ROS and RNS, according to the manufacturer. In our preliminary experiments, reactive species were detected under a fluorescence microscope after CAP treatment for up to 3 min, and the number of reactive species detected increased as the treatment duration increased (Figure 2d). To determine the CAP effectors responsible for the additive anticancer effect with an EGFR inhibitor, A375 cells were treated with ROS-generating H_2_O_2_ or RNS-producing nitroprusside (SNP), alone or in combination with CAP. As concentrations of H_2_O_2_ and SNP greater than 200 µM and 400 µM, respectively, were highly cytotoxic (Figure 2e,g), we selected H_2_O_2_ at 200 µM and SNP at 400 µM for the following experiments. Both H_2_O_2_ and SNP showed additive anticancer effects when combined with an EGFR inhibitor on A375 cells (Figure 2f,h), suggesting that the additive anticancer effect of CAP and an EGFR inhibitor was at least partially due to these reactive species.

### 2.3. In Vivo Anticancer Effects of Cold Atmospheric Plasma

**Figure 3:**
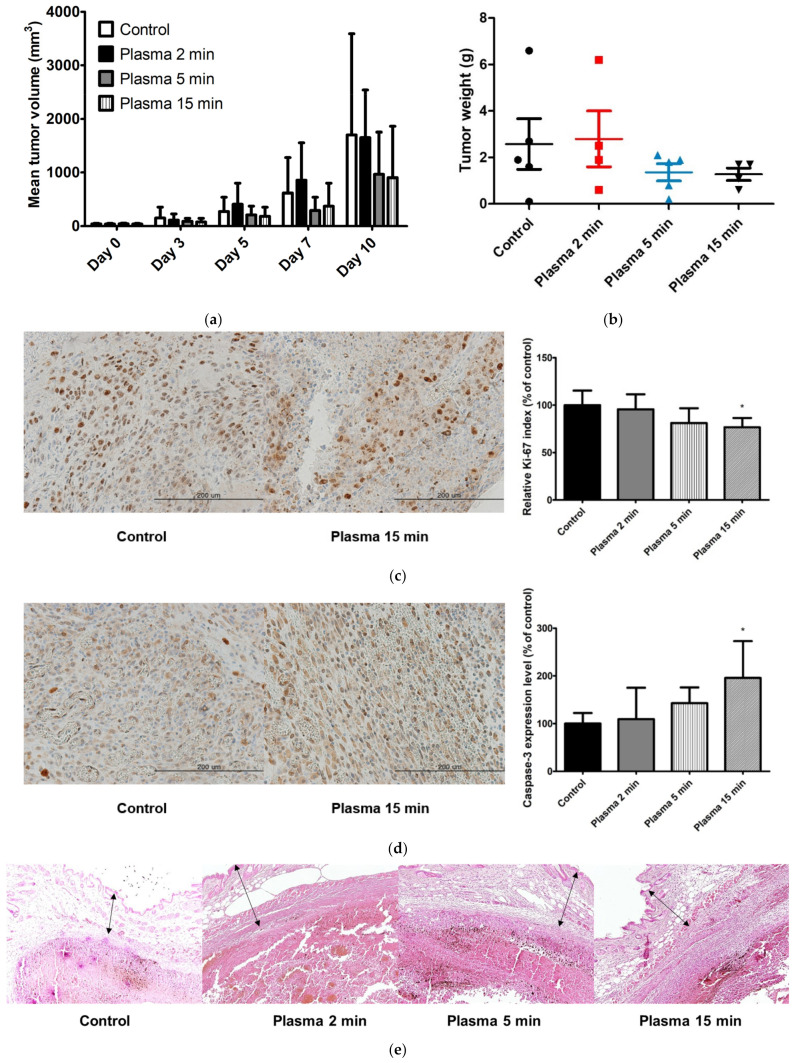
Anticancer effect of cold atmospheric plasma (CAP) in a syngeneic mouse model with B16F10 melanoma cells. After the growth of tumors, five mice were each treated five times, once every other day (i.e., on days 0, 2, 4, 6, and 8), with CAP for 2 min, 5 min, or 15 min, whereas five untreated mice served as controls. (**a**) Tumor volumes on days 0, 3, 5, 7, and 10. Tumor sizes were measured using calipers, and tumor volumes were calculated as (length × width^2^)/2. (**b**) Tumor weights were measured on day 10. (**c**) Representative images of immunohistochemical labeling of Ki-67 in control and 15 min plasma treatment groups on day 10 (Ki-67, ×400). The Ki-67 indices of left and mid panel were assessed as 38.1% and 24.4%, respectively. The mean value of Ki-67 index ± standard deviation in CAP-treated mice relative to control is shown in the right panel. * *p* < 0.05. (**d**) Representative images of immunohistochemical labeling of caspase-3 in control and 15 min plasma treatment groups on day 10 (caspase-3, ×400). The mean expression level of caspase-3 ± standard deviation in CAP-treated mice relative to control is shown in the right panel. * *p* < 0.05, ** *p* < 0.01, *** *p* < 0.001. (**e**) Representative histological images of B16F10 melanomas on day 10 from a control mouse and from mice treated with CAP for 2 min, 5 min, and 15 min (hematoxylin and eosin, ×100). Arrows indicate preserved normal structures overlying the tumors.

The anticancer effects of CAP treatment were evaluated using syngeneic mouse models. Twenty mice were inoculated with B16F10 melanoma cells, and 18 with MC38 colon cancer cells. Following the growth of B16F10 melanomas, five mice were each treated five times, once every other day (i.e., on days 0, 2, 4, 6, and 8), with CAP for 2 min, 5 min, or 15 min. Five untreated mice served as controls. Tumor volumes were measured on days 0, 3, 5, 7, and 10. Additionally, tumor growth inhibition (TGI) was calculated, and tumor weights were measured on day 10. Mean tumor volumes on day 0 did not differ significantly, being 38.82, 37.62, 40.05, and 36.35 mm^3^ in the control group and mice treated with CAP for 2 min, 5 min, and 15 min, respectively. CAP treatments for 5 min and 15 min reduced tumor volumes and weights on day 10 (Figure 3a,b, Supplementary Figure S1). TGIs in mice treated with CAP for 2 min, 5 min, and 15 min were 3.0%, 43.4%, and 47.0%, respectively. In the immunohistochemical studies, the Ki-67 index decreased as the duration of CAP treatment increased, suggesting that the cellular proliferation assessed by Ki-67 can be decreased by CAP treatment (Figure 3c) [27]. In contrast, the expression level of caspase-3 increased as the duration of CAP treatment increased, which means that CAP treatment can induce apoptosis in a dose-dependent manner (Figure 3d). Histological examination showed the preserved overlying epidermal and dermal layers in both the control group and the CAP-treated groups (Figure 3e).

**Figure 4:**
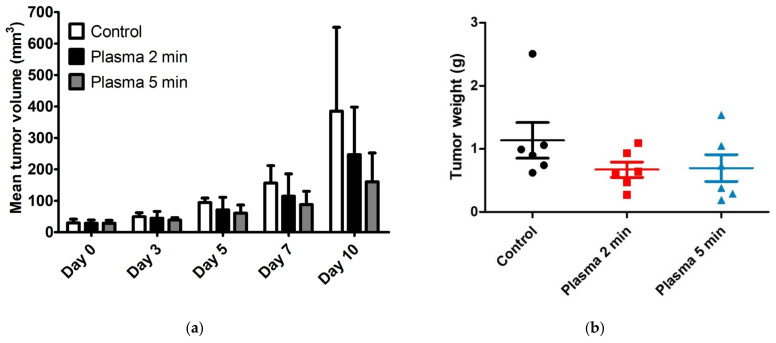

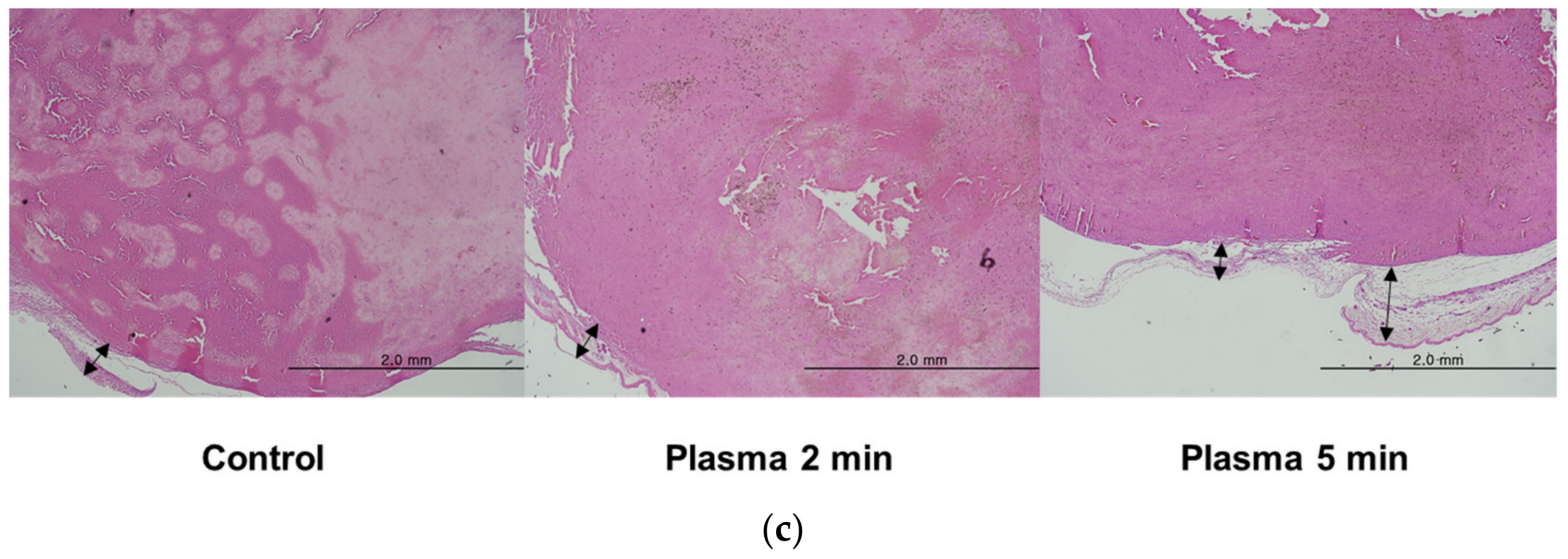
Anticancer effects of cold atmospheric plasma (CAP) in a syngeneic mouse model with MC38 colon cancer cells. After the growth of tumors, six mice were each treated five times, once every other day (i.e., on days 0, 2, 4, 6, and 8), with CAP for 2 min or 5 min, whereas six untreated mice served as controls. (**a**) Tumors were measured on days 0, 3, 5, 7, and 10. Tumor sizes were measured using calipers, and tumor volumes were calculated as (length × width^2^)/2. (**b**) Tumor weights were measured on day 10. (**c**) Representative histological images of MC38 colon tumors on day 10 from a control mouse and from mice treated with CAP for 2 min and 5 min (hematoxylin and eosin, ×40). Arrows indicate preserved normal structures overlying the tumors.

In the MC38 colon cancer model, 18 mice were inoculated with MC38 colon cancer cells. Following tumor growth, six mice were each treated five times, once every other day (i.e., on days 0, 2, 4, 6, and 8) with CAP for 2 min or 5 min, while six untreated mice served as controls. Tumor volume and weight reduction did not differ markedly between B16F10 melanoma inoculated mice treated with CAP for 5 min and 15 min, so the latter time point was omitted from the MC38 colon cancer model. Mean tumor volumes on day 0 did not differ significantly, being 29.53, 28.57, and 28.39 mm^3^ in control mice and mice treated with CAP for 2 min and 5 min, respectively. As in the B16F10 melanoma model, tumor volumes were measured on days 0, 3, 5, 7, and 10, TGI was calculated, and tumor weights were measured on day 10. CAP treatments for 2 and 5 min reduced tumor volumes and weights on day 10 (Figure 4a,b, Supplementary Figure S2). TGIs in mice treated with CAP for 2 and 5 min were 35.8% and 58.3%, respectively, relative to control mice. Histopathological examination revealed the preserved overlying epidermal and dermal layers in both the control group and the CAP-treated groups (Figure 4c).

## 3. Discussion

The clinical application of CAP requires an understanding of its anticancer potency in vivo and in vitro. The present study, therefore, assessed the anticancer effects of CAP applied directly to various cancer cell lines in vitro and to tumors in syngeneic mice inoculated with melanoma and colon cancer cell lines.

There was little effect on the viability of NHMs following CAP treatments for up to 150 sec. In contrast, the viability of melanoma and colon cancer cell lines was significantly reduced by CAP treatment, demonstrating that CAP has selective anticancer effects. The mechanisms underlying the greater potency of CAP against cancer cells than against nonmalignant cells and the differences in the degree of sensitivity of cancer cells to CAP were under active investigation [28]. These differences may be due to differences in ROS levels after CAP treatment [29,30]. Basal intracellular ROS levels are higher in cancer cells than in nonmalignant cells because malignant cells generally have a stronger metabolism [31]. Therefore, an additional influx of ROS in response to CAP treatment can result in ROS levels that cannot be tolerated by cancer cells [32]. Differences in the levels of expression of aquaporins may also contribute to differences in intracellular ROS levels [13]. Aquaporin expression was higher in the cytoplasmic membranes of malignant cells than in nonmalignant cells [33]. Higher expression of those channels may result in faster diffusion of CAP-derived H_2_O_2_, activating the downstream pathway leading to cellular apoptosis [13]. Similarly, the intracellular antioxidant system can contribute to differences in sensitivity to CAP between cell lines [34].

Apoptosis is the active and physiological form of cell death involving the destruction of cellular components. In contrast, necrosis is passive and accidental cell death caused by sudden environmental change [35]. The results of the present study suggest that the anticancer effects of CAP are mediated by cellular apoptosis; cleaved caspase-3—a key factor for apoptosis—was increased by CAP exposure in a treatment-duration-dependent manner. Members of the Bcl-2 family control the intrinsic pathway of apoptosis [36]. Given that the expression of Bcl-2 was not altered by CAP, the CAP-induced anticancer effect may involve the extrinsic pathway of apoptosis. Nrf2 is a transcription factor regulating genes involved in antioxidant responses, including HO-1 [37]. The expression level of Nrf2 was not altered 24 h after CAP treatment, which can possibly be attributed to its constitution as a short-lived protein and upstream regulator of HO-1 [38].

EGFR is overexpressed in many cancers. Although EGFR expression and its role in melanoma are conflicting, the clinical significance of EGFR has been suggested in the literature, especially in advanced or distant metastatic melanoma, a debilitating cutaneous cancer with aggressive behavior and a poor prognosis [39]. EGFR protein expression is a marker of melanoma metastasis [40], and a prognostic factor for survival outcomes [41]. The present study demonstrated that the administration of an EGFR inhibitor could intensify the antimelanoma effect of CAP. The findings also indicated that ROS and RNS generated by CAP are responsible for the additive antimelanoma effect of a combination of CAP and an EGFR inhibitor. This potential combination may play a role in future antimelanoma therapy, especially when the antimelanoma effect of CAP alone is not sufficient.

In vivo experiments also showed that CAP treatment had tumoricidal effects. The degree of TGI, however, differed in the two cancer models tested. In contrast to the in vitro results showing that B16F10 cells were more sensitive to CAP treatment than MC38 cells, TGI in response to the same duration of CAP treatment was higher in MC38 colon cancer than in B16F10 melanoma cells. CAP treatments for 2 min in each case effectively reduced the growth of MC38 colon cancer cells but had little effect on the growth of B16F10 melanoma cells. However, growth rates were much higher in tumors induced by B16F10 melanoma than in those induced by MC38 colon cancer cells. The mean volumes of B16F10 melanoma in the control group on days 0 and 10 were 38.82 and 1700.62 mm^3^, respectively, whereas the mean volumes of MC38 colon cancers in the control group on days 0 and 10 were 29.53 mm^3^ and 384.91 mm^3^, respectively.

Consequently, the mean duration of CAP exposure per tumor volume was much higher in the MC38 colon cancer model. These results demonstrate the existence of a gap between in vitro and in vivo studies, reinforcing the requirement for more in vivo experiments before treating patients with CAP. Indeed, sensitivity to CAP at the cellular level does not necessarily indicate good responses to CAP in vivo or in clinical trials. Other factors such as tumor volume and growth rate should be considered. Increasing the duration of exposure to CAP from 5 min to 15 min had little effect on the B16F10 melanoma model, suggesting that the ability of CAP to inhibit tumor growth and the propensity toward tumor growth had reached equilibrium. Therefore, a combination of direct and indirect exposure to CAP using CAP-stimulated solutions should be considered when treating large-volume tumors, as the depth of effective tissue penetration by directly applied CAP can be limited [42,43]. As we utilized syngeneic mouse models in which a functional immune system was present, immune cells constituted the tumor microenvironment [44]. These immune cells can actively participate in the anticancer effect of CAP, which may also have contributed to the discrepancy between the results of the in vitro and the in vivo experiments [45]. CAP treatment can expose new antigens and activate the immunogenic cell death pathway, mediated by reactive species. In addition, by reducing the immunosuppressive cell population in the tumor microenvironment, CAP can have an additional beneficial effect [46]. Future studies elucidating the effect of CAP on the tumor microenvironment, including immune cells, would be of interest.

In addition, CAP treatment effectively decreased cellular proliferation and induced apoptosis in a dose-dependent manner as shown in the immunohistochemical studies using antibodies including Ki-67 and caspase-3. The results were in line with previously reported effects of CAP on other cancer cell lines [27,47]. Direct CAP treatment was also found histologically to have a selective anticancer effect in the B16F10 melanoma and the MC38 colon cancer model, because exposure to CAP for up to 15 min had little effect on normal adjacent tissue. These findings indicated that CAP treatment was relatively safe and that physical factors, such as thermal effect, radiation, and electromagnetic fields, played minor roles in the interactions between CAP and tissues. The effect of physical factors on the in vivo application of CAP is largely unknown. However, the increased temperature of CAP-treated tumor tissue had a negligible effect [48]. In the MC38 colon cancer model, however, tumors were located too superficially to assess the selectivity of CAP treatment.

## 4. Materials and Methods

### 4.1. Experimental System

The CAP device employed in this study was the MediPL^®^ plasma torch system (2.5 W, argon flow, 2.0 L/min) developed and built by MediPL, Inc., Seoul, Republic of Korea.

### 4.2. Cell Cultures

NHMs were obtained from Invitrogen (Carlsbad, CA, USA) and cultured in Medium 254, supplemented with Human Melanocyte Growth Supplement (Thermo Fisher Scientific, Inc., Waltham, MA, USA) at 37 °C and 5% CO_2_. NHMs were used in passages between 3 and 7. B16F10 murine melanoma cells were obtained from the Korean Cell Line Bank (Seoul, Republic of Korea). Human melanoma cells (A375, A2058) and colon cancer cells (MC38) were purchased from the American Type Culture Collection (Manassas, VA, USA). These cells were maintained in Dulbecco’s Modified Eagle’s Medium (DMEM), supplemented with 10% fetal bovine serum (FBS) and 1% (*v*/*v*) penicillin–streptomycin in 5% CO_2_ at 37 °C.

### 4.3. Cell Viability Assay

**Figure 5:**
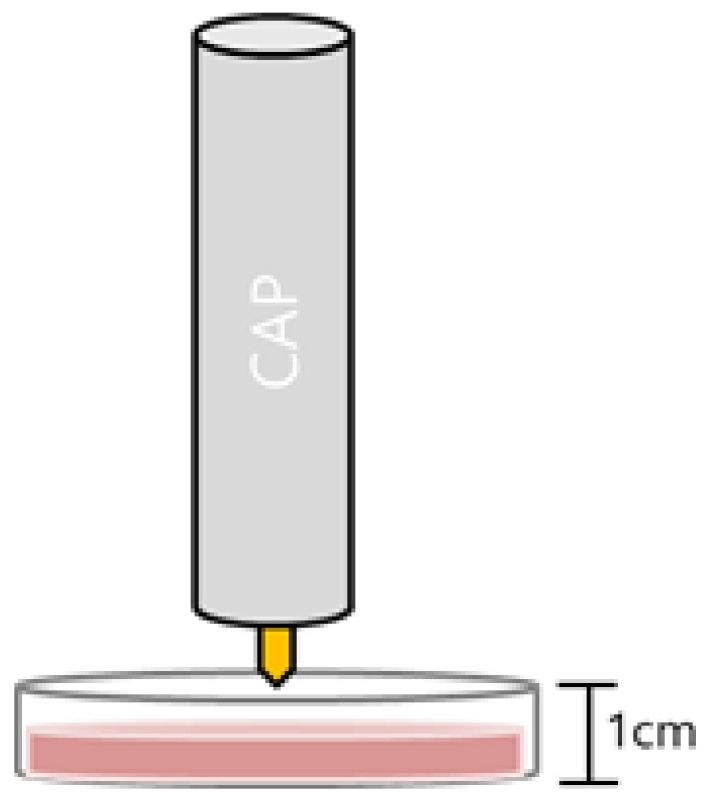
Schematic diagram of the cold atmospheric plasma (CAP) device placed just over the culture medium.

The viability of NHMs, B16F10, A375, A2058, and MC38 cells on 35 mm culture plates was measured 24 h after CAP treatment for the indicated periods for crystal violet assays according to the standard protocols recommended by the manufacturer (Sigma-Aldrich, St. Louis, MO, USA). Briefly, cells were stained with 0.1% crystal violet in 10% ethanol for 5 min at room temperature and rinsed four times with distilled water. The crystal violet retained by adherent cells was extracted with 95% ethanol, and the absorbance at 590 nm was determined using a microplate reader (Molecular Devices, Sunnyvale, CA, USA). Relative cell viability was calculated by dividing the absorbance of the experimental group by the absorbance of the control group. The anticancer effects of combinations of an EGFR inhibitor and CAP were determined by pretreating cells with the EGFR inhibitor AG1478 (Sigma-Aldrich) for 1 h, followed by treatment with CAP for 120 sec and assessment of cell viability after 24 h. After the CAP device was placed as close as possible to the culture medium, the cells were treated with CAP directly (Figure 5). The effects of combinations of the EGFR inhibitor AG1478 with H_2_O_2_ (Sigma-Aldrich) and SNP (Sigma-Aldrich) on cancer cell viability were assessed by pretreating cells with AG1478 for 1 h followed by treatment with H_2_O_2_ or SNP, and assessment of cell viability after 24 h.

### 4.4. Western Blot Analysis

Western blot analysis to assess the levels of protein expression 24 h after treatment of B16F10 and MC38 cells seeded in 35 mm culture plates with CAP was performed using antibodies against Bcl-2, cleaved caspase-3, HO-1, Nrf2 (Cell Signaling, Danvers, MA, USA), and β-actin (Sigma-Aldrich), the last-mentioned of which was used as an internal loading control. Levels of protein expression were quantified by densitometry after normalization to the optical density of β-actin using ImageJ 1.52 software (National Institute of Health, Bethesda, MA, USA).

### 4.5. Quantitative Real-Time Polymerase Chain Reaction (qPCR)

A375, B16F10, A2058, and MC38 cells were cultured in 12-well plates for 24 h, and total cellular RNA was extracted from the cells using a FavorPrepTM Total RNA Purification Mini Kit according to the manufacturer’s instructions (Favorgen, Ping Tung, Taiwan). The quantity and quality of the isolated RNA were determined using a NanoDrop^®^ ND-1000 Spectrophotometer (ND-1000, NanoDrop Technologies, Wilmington, DE, USA). Single-stranded cDNA was synthesized from 1 μg of total RNA using a Revert Aid First Strand cDNA Synthesis Kit according to the manufacturer’s instructions (Thermo Scientific, Rockford, IL, USA). Quantitative real-time PCR was performed using the LightCycler^®^ 480II thermal cycler coupled with SYBR Green chemistry (Roche Applied Science, Penzberg, Germany). The amplification protocol consisted of an initial denaturation at 95 °C for 5 min, followed by 55 cycles of denaturation at 95 °C for 10 s, annealing at 60 °C for 10 s, and extension at 72 °C for 10 s. Amplification primers for EGFR included 5′-CCCACTCATGCTCTACAACCC-3′ (forward) and 5′-TCGCACTTCTTACACTTGCGG-3′ (reverse).

### 4.6. Intracellular Reactive Oxygen Specieis Measurement

B16F10 cells were cultured in 96-well plates for 24 h and rinsed with phosphate buffered saline (PBS). The cells were cultured with 1 uM CM-H2DCFDA (5-(and-6)-chloromethyl 2′,7′ dichloro-dihydro fluorescein diacetate, acetyl ester, Reactive Oxygen Species Detection reagents, invitrogen, C6827, Eugene, OR, USA) containing PBS for 30 min, 4 h after treatment with CAP or H_2_O_2_ 100 μM. Fluorescence intensity for ROS was then detected using a microplate reader (VICTOR X2, PerkinElmer, Waltham, MA, USA).

### 4.7. In Vivo Experiments

All animal experiments were performed in compliance with the Principles of Laboratory Animal Care, formulated by the Institutional Animal Care and Use Committee (IACUC) of the Asan Institute for Life Sciences, Asan Medical Center, Seoul, Republic of Korea, and were consistent with the guidelines of the Institute of Laboratory Animal Resources (ILAR).

Thirty-eight C57BL/6 mice were purchased from ORIENT BIO, Inc. (Seongnam, Republic of Korea). At age 6 weeks, their fur was removed using small animal clippers, and 5 × 10^5^ B16F10 or MC38 cells were intradermally injected into each mouse using a 2.5 μL Hamilton syringe (Hamilton, Reno, NV, USA). The mice were followed-up routinely, and tumor size was measured throughout the study period using calipers. Tumor volume was calculated as (length × width^2^)/2. The initial tumor-size measurement was performed 7 days after the intrdermal injection of cancer cells (day 0). For CAP treatment, the mice were anesthetized using isoflurane; CAP was applied 1 ± 2 mm above the skin for the designated time while continuously moving the probe to cover the entire tumor, with 0.5 cm margins. TGI was defined as the difference between the mean tumor volumes of treated and control groups, with the % TGI calculated as % TGI = (mean tumor volume of the control group—mean tumor volume of the treated group)/mean tumor volume of the control group × 100. On day 10, the tumors were excised to measure their weight, and skin samples were obtained for histological evaluation.

### 4.8. Histological and Immunohistochemical Studies

The skin samples from the in vivo experiments were fixed in 10% paraformaldehyde (in 0.1 M PBS, pH 7.4), mounted in paraffin, and sectioned into 5 μm thick paraffin sections, which were stained with hematoxylin and eosin.

Immunohistochemical staining for Ki-67 and caspase-3 was performed using a Benchmark XT immunostainer (Ventana Medical Systems, Roche, Basel, Switzerland). The resected tissue specimens were fixed in 10% formaldehyde, embedded in paraffin, and cut into 4 μm thick sections that were deparaffinized with xylene, dehydrated with ethanol. For antigen retrieval, the sections were boiled in Tris/Borate/EDTA buffer (pH 8.4) for 60 min. After cooling to room temperature, the sections were washed in Tris buffer (pH 7.6), and then they were incubated with 3% hydrogen peroxide for 4 min. Next, the sections were incubated with primary antibodies against Ki-67 ((ab16667) diluted 1:100; abcam, Waltham, Boston) or caspase-3 ((9662) diluted 1:1000; Cell Signaling, Danvers, MA, USA) for 36 min at room temperature. After washing in Tris buffer (pH 7.6), the sections were subjected to an UltraMap anti-Rb HRP (Roche, Basel, Switzerland) for 12 or 16 min at room temperature. After washing in Tris buffer (pH 7.6), the peroxidase reaction was developed with an ultraView Universal DAB Detection Kit (Roche, Basel, Switzerland) for 8 min at room temperature. The sections were washed in Tris buffer (pH 7.6), and the sections were counterstained with hematoxylin and bluing reagent (Roche, Basel, Switzerland). The sections were then dehydrated, treated with xylene, and coverslipped.

The Ki-67 index was defined as the percentage of cells with Ki-67 positive nuclear immunostaining. At least three representative areas from each case were selected, and microphotographs were taken at ×400. At least 500 cells for an area were manually counted, and the average fraction of Ki-67 positive cells were calculated. For the quantitative assessment of caspas-3 expression level, at least three representative areas for each case were photographed at ×400. The expression level was then calculated by measuring the fraction of stained area using Image J (National Institute of Health, Bethesda, MA, USA).

### 4.9. Statistical Analysis

The statistical significance of the differences was assessed using analysis of variance (ANOVA), followed by the Student’s *t*-test. *p*-values of <0.05 were considered statistically significant. All statistical analyses were performed using R version 3.5.3 (R Foundation for Statistical Computing) software.

## 5. Conclusions

This study demonstrated that direct CAP treatment could have selective anticancer activity in vitro and in vivo. Direct application of CAP in vivo effectively inhibited B16F10 tumor growth by 50% and MC38 tumor growth by more than 50%, suggesting that CAP effectively inhibited the growth of these types of cancers. Direct CAP treatment can be used as adjuvant or neo-adjuvant therapy to reduce tumor burden before or after primary resection and for palliative purposes. In addition, a combination of an EGFR inhibitor and CAP may be a candidate for treating a subset of melanomas, including more aggressive forms.

## Data Availability

The data that support the findings of this study are available from the corresponding author upon reasonable request.

## Supplementary Materials

The following supporting information can be downloaded at: https://www.mdpi.com/article/10.3390/molecules28104171/s1. Figure S1: Anticancer effect of cold atmospheric plasma in a syngeneic mouse model with B16F10 melanoma cells; Figure S2: Anticancer effects of cold atmospheric plasma in a syngeneic mouse model with MC38 colon cancer cells.

## Sample Availability

Sample is not available due to the restriction of resources.
