# Supplementary material for: Anticancer Effect of Cold Atmospheric Plasma in Syngeneic Mouse Models of Melanoma and Colon Cancer
Source: Molecules. 2023 May 18;28(10):4171. doi: 10.3390/molecules28104171 (PMC10224096; doi:10.3390/molecules28104171)
Supplement: Supplementary file 1 [file molecules-28-04171-s001.zip › molecules-2200544-supplementary.pdf]

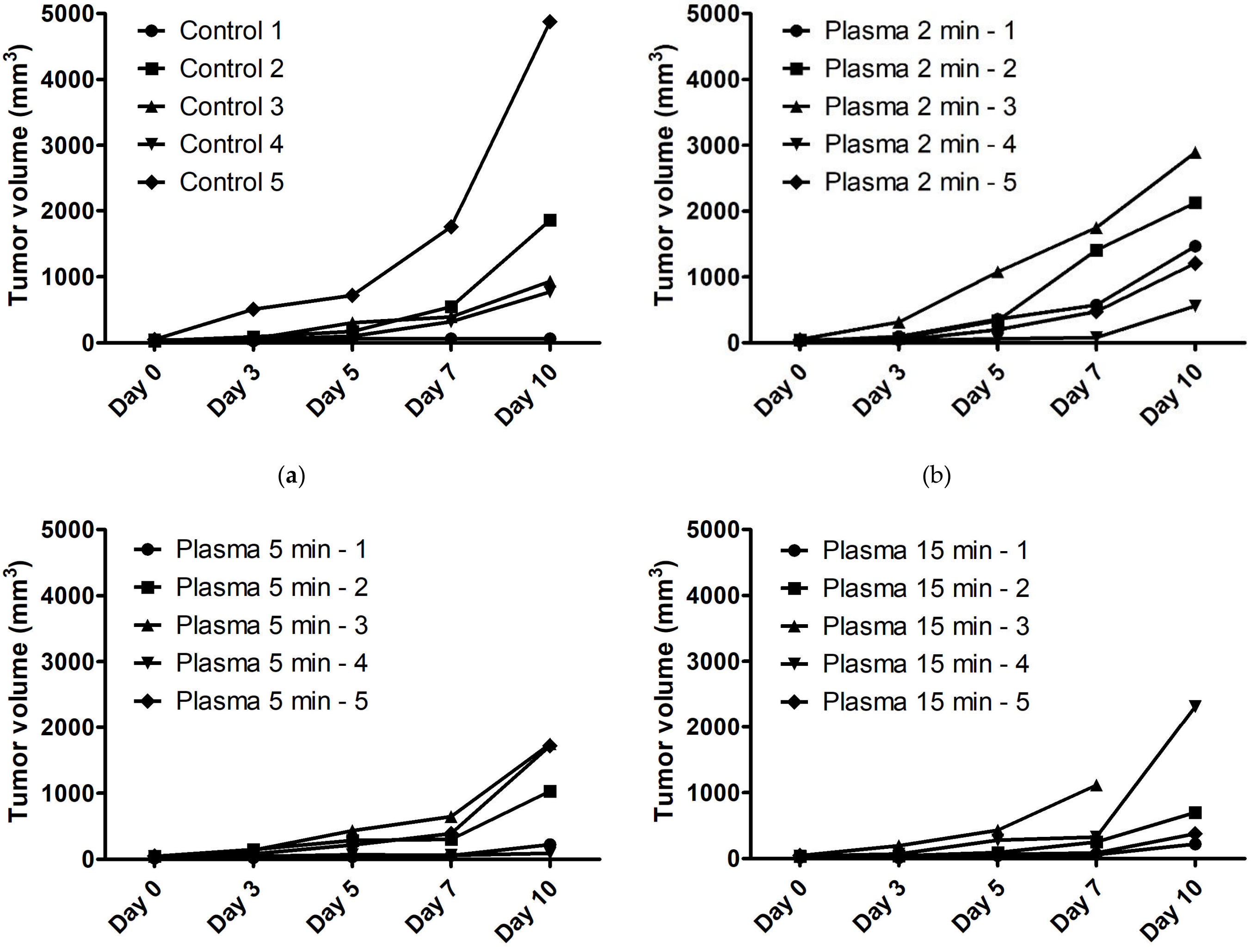

(**a**) (b)

(**c**) (**d**)

Supplementary Figure S1. Anti-cancer effect of cold atmospheric plasma (CAP) in a syngeneic mouse model with B16F10 melanoma cells. After the growth of tumors, five mice each were treated five times, once every other day (i.e., on days 0, 2, 4, 6, and 8), with CAP for 2 min, 5 min, or 15 min, whereas five untreated mice served as controls. Tumor volume of the individual mouse in the control group (**a**), CAP 2 min (**b**), CAP 5 min (**c**), and CAP 15 min (**d**) treated groups.

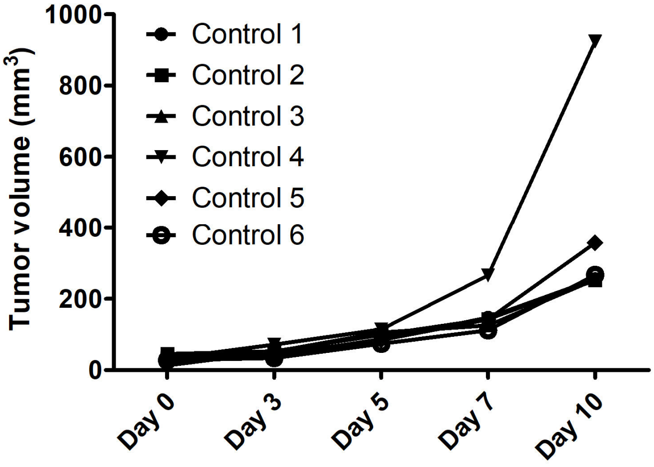

(**a**)

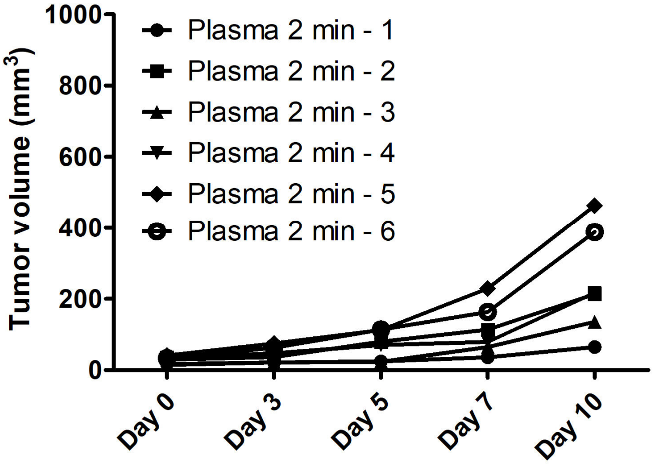

(b)

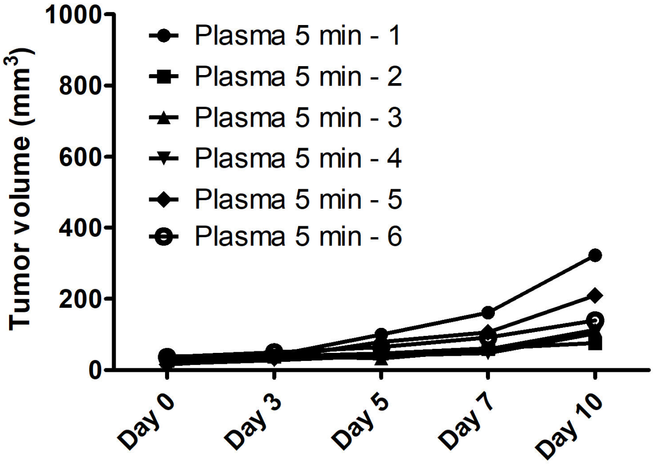

(**c**)

Supplementary Figure S2. Anti-cancer effects of cold atmospheric plasma (CAP) in a syngeneic mouse model with MC38 colon cancer cells. After the growth of tumors, six mice were each treated five times, once every other day (i.e., on days 0, 2, 4, 6, and 8), with CAP for 2 min or 5 min, whereas six untreated mice served as controls. Tumor volume of the individual mouse in the control group (**a**), CAP 2 min (**b**), and CAP 5 min (**c**) treated groups.
